# Nanostructured Polystyrene Doped with Acetylsalicylic Acid and Its Antibacterial Properties

**DOI:** 10.3390/ma13163609

**Published:** 2020-08-14

**Authors:** Dominik Fajstavr, Klára Neznalová, Nikola Slepičková Kasálková, Silvie Rimpelová, Kateřina Kubičíková, Václav Švorčík, Petr Slepička

**Affiliations:** 1 Department of Solid State Engineering, University of Chemistry and Technology Prague, 16628 Prague, Czech Republic; dominik.fajstavr@vscht.cz (D.F.); klara.neznalova@vscht.cz (K.N.); nikola.kasalkova@vscht.cz (N.S.K.); kubicikk@vscht.cz (K.K.); vaclav.svorcik@vscht.cz (V.Š.); 2 Department of Biochemistry and Microbiology, University of Chemistry and Technology Prague, 16628 Prague, Czech Republic; silvie.rimpelova@vscht.cz

**Keywords:** polystyrene, acetylsalicylic acid, excimer laser, LIPSS, surface morphology, antibacterial properties

## Abstract

Homogeneous polystyrene foils doped with different concentrations of acetylsalicylic acid were prepared by the solvent casting method. The surface morphology and surface chemistry of as-prepared foils were characterized in detail. Excimer laser (krypton fluoride, a wavelength of 248 nm) was used for surface nanopatterning of doped polystyrene foils. Certain combinations of laser fluence and number of laser pulses led to formation of laser-induced periodic surface structures (LIPSS) on the exposed surface. Formation of the pattern was affected by the presence of a dopant in the polystyrene structure. Significant differences in surface chemistry and morphology of laser-treated foils compared to both pristine and doped polystyrene were detected. The pattern width and height were both affected by selection of input excimer exposure conditions, and the amount of 6000 pulses was determined as optimal. The possibility of nanostructuring of a honeycomb-like pattern doped with acetylsalicylic acid was also demonstrated. Selected nanostructured surfaces were used for study the antibacterial properties for a model bacteria strain of *S. aureus*. The combination of altered surface chemistry and morphology of polystyrene was confirmed to have an excellent antibacterial properties.

## 1. Introduction

It has been reported that during polarized pulsed laser modification with laser fluence values under the ablation threshold, a wrinkled structure was formed on a polymeric material [1]. This thermal physical process is known in the literature as a so-called laser-induced periodic surface structuring (LIPSS). The mechanism of this formation is based on the interference of both the light scattered on the surface and the incident light. That creates a distribution of laser energy on the substrate surface with the areas of higher and lower temperatures according to the absorbance of the polymer at laser wavelengths [2]. The main driving force for the polymer surface to flow from areas with higher temperatures to lower temperatures and form periodic structures is the temperature gradient [3]. As mentioned above, the main condition for LIPSS formation on a polymeric surface is its absorbance of laser light in the corresponding wavelength. On the other hand, studies and works are focusing on LIPSS formation in non-absorbing materials [4]. A two-step procedure, containing LIPSS formation on stainless steel and further replication of the structures onto biomaterials using the steel LIPSS as a scaffold, has been devised by Hendrikson et al. for stem cell biology and applications in regenerative medicine [5].

Another way to prepare LIPSS structures on non-absorbing materials is to use femtosecond laser pulses. In this case, ionization and multiphoton absorption processes mediate the coupling of modifying laser light with the polymeric material due to the high intensities involved [6,7,8]. As a result, with this approach, we can achieve LIPSS formation on the polymers that is transparent for the ultraviolet (UV) region. The next advantage of the femtosecond laser is a wider range of morphology and size control of periodic structures. The wavelength of the laser is closely related to the periodic ripple structures [9]. To achieve specific morphology on the surface, several factors need to be taken into account, such as the type of a substrate, diffusivity, thermal conductivity, and the absorption of the polymer [10,11].

The important factor of the polymeric material is its molecular weight (M_w_) as it defines its physical properties, e.g., mechanical properties and transition temperatures [12]. During the last few years, we have observed increased interest in laser ablation techniques and studies. However, only several studies reported about M_w_ influence on polymer properties [13,14,15,16,17,18,19], even though the knowledge of M_w_ values and their effect on polymeric ablation is crucial for laser processing applications. A study focusing on morphological changes of polymethylmethacrylate (PMMA) and (polystyrene) PS of different M_w_ brings Masuhara et al. [15]. There, the behavior of systems with various M_w_ values after laser irradiation, which showed different rates of expansion and contraction of the polymer films, is investigated. This behavior is directly related to the characteristic length of the polymer chain and its transition temperatures (T_g_). Procedure of preparing LIPSS structures on the surface of composite substrates based on polymer-metal or polymer-graphene are publish in various articles [20,21]. The laser modification leads to the formation of regular structures, but together with the change in the M_w_ of the polymer, the threshold of the laser fluence values required for the preparation of the LIPSS also changes. Understanding the dependence of M_w_ on polymer properties is necessary for laser processing applications especially of low absorbing polymer (e.g., laser deposition, tissue processing, etc.) [22].

In biomedical research, the treatment of the polymeric surface plays a significant role in enhanced biocompatibility with preservation of the bulk material and in targeted drug release [22]. Other desired effects achieved by treatment of the polymeric surface are improved cytotoxic and antibacterial properties with their use in bioengineering. Reconstructive medicine uses these effects of surface-modulated polymeric biomaterials for improved absorption, enhanced functionality, and attachment of bioactive compounds to surfaces. Laser-induced periodic surface structuring methods are widely used there, where optimal structures and controlled topography need to be designed [23]. The study of Heitz et al. have demonstrated a new method for enhanced proliferation of human vascular cells on UV-treated poly-tetrafluoroethylene (PTFE) polymer along with increased adhesion [24]. Other articles report improved adhesive properties of nanoparticles and their integration into the polymeric surface after polymer UV-laser treatment [25].

The effect of doping polymeric material with the drug substance lecithin is described in the work of Lu et al. [26]. If small molecules were added to the polymer matrix, the transition temperature (T_g_) of the polymer decreased because of the plasticization effect of doped molecules on the polymer chains. Laser-induced periodic surface structuring can be prepared on the polymer surface at lower energy values, at which the effect of different doping levels of lecithin on the formation of LIPSS on a polyimide (PI) film is investigated. In PI film doped with lecithin there is a confirmed fall of energy values needed to produce LIPSS in comparison to non-doped samples. The results reveal that the lecithin molecules tend to migrate to the surface of the polymer after laser modification [26].

However, to our best knowledge, there is no study focusing on LIPSS formation on polymeric substrate doped with acetylsalicylic acid. Acetylsalicylic acid (ASA), commonly known as aspirin, is one of the most important anti-inflammatory drugs in the world. It is prescribed to relieve minor pains and to fever reduction [27]. Another application for ASA according to several studies is to treat coronary heart disease, antithrombotic, pregnancy-induced pre-eclampsia, and prevent colon cancer [28,29]. However, this massive use of aspirin has resulted in problems of overdosing [30]. In this research article we have focused on the preparation of doped polystyrene films with ASA and subsequent exposure with an excimer laser. The changes in surface morphology and surface chemistry thus influencing the surface wettability were described in detail. Selected foils were used as antibacterial materials with outstanding results. Compared with paper [20] the graphene nanoplatelets in [20] were not dissolved in the solution and a composite was formed instead. The acetylsalicylic acid is fully dissolved in the polymer/solvent system, thus the major character of primary polymers is different. The major idea was to study the influence of ASA, laser treatment or their combination on antibacterial properties. The goal of antimicrobial film construction was successfully achieved by the combination of appropriate ASA dotation and excimer exposure.

## 2. Materials and Methods

### 2.1. Materials and Chemicals

A commercial polystyrene foil (PS; 50 μm thickness, biaxially oriented, density of 1.05 g·cm^−3^, received from Goodfellow Cambridge Ltd. (Ermine Business Park, Huntingdon, UK). ASA from Sigma Aldrich (purity > 99.0) was used. The polystyrene foil was diluted in 50 mL of chloroform, which was continuously stirred on magnetic stirrer for one hour. Additionally, appropri.ate amount of ASA was added so that the weight ratio was set to 5% and 10%, respectively. The solution was continuously stirred for one more hour. The solution was transferred into the Petri dish and evaporated under RT, than it was removed and used a substrate for excimer exposure. The advantage of polystyrene application is that it exhibits a good biocompatibility, as in previous experiments has been used also for cell growth guidance [31].

### 2.2. Surface Exposure of Substrates

Surface structures were also modified with a pulsed excimer KrF laser (Coherent Inc., Santa Clara, CA, USA) COMPexPro 50 F, with a wavelength of 248 nm and a pulse duration of 20–40 nanoseconds, repetition rate of 10 Hz). The laser beam was linearly polarized with a prism polarizer (size 25 × 25 × 25 mm^3^, fused quartz) with an active polarization layer (model PBSO-248-100). For uniform irradiation of the substrate, a screen was inserted here, which transmits only the central part of the laser beam (0.5 × 1.0 cm^2^) with a homogeneous energy distribution. The amount of 6000 laser pulses was used to achieve uniformity of the surface structure. The laser fluence value was set between 0–20 mJ·cm^−2^.

### 2.3. Substrate Characterization

The surface morphology, roughness and area of the samples were determined by atomic force microscopy (AFM) using the Dimension ICON (Bruker Corp, Billerica, MA, USA). The samples were analyzed in Scan-Assyst mode using nitride lever SCANASYST-AIR with Si tip (spring constant of 0.4 N·m^−1^). NanoScope Analysis software was applied for data processing. The surface topography was also determined by the scanning electron microscope (SEM) using LYRA3 (Tescan, Brno, Czech Republic). The applied acceleration voltage for SEM was 10 kV. The substrates were covered with the Pt conductive layer of 20 nm thickness by a diode sputtering method (Quorum Q300T, Quorum Technologies Ltd., Laughton, East Sussex, UK).

The elemental composition on the sample surface was analyzed by X-ray photoelectron spectroscopy (XPS) using spectrometer ESCAProbeP (Omicron Nanotechnology, Taunusstein, Germany). The source was monochromatic X-ray at energy of 1486.7 eV. Atomic concentrations of elements were determined from the individual peak areas using CasaXPS software. Measurements were undertaken on the equipment of Omicron Nanotechnology; the primary X-ray beam was monochrome radiation of Al lamp with energy 1486.7 eV. Constant analyser energy (CAE) mode was used, intensity calibration was solved on the base of former measurements of copper and from copper spectra-derived calibration constants. Measured spectra were evaluated by software CasaXPS where after intensity calibration the area of peaks and relative sensitivity factors (RSF) from the database were used for the determination of concentrations.

The wettability of the polymer surface was studied goniometrically by measuring the contact angle. The contact angle was measured by the Surface Energy Evaluation System (SEE System, Advex Instruments, Brno, Czech Republic); 8 drops of 8.0 ± 0.2 µL volume of distilled water were applied to the sample by an automatic pipette and the consequent photographs were evaluated.

### 2.4. Antibacterial Activity

The antibacterial properties of pristine PS, PS doped with ASA, and doped samples subsequently treated with excimer laser were evaluated using the bacterial strain of Gram-positive *S. aureus* (CCM 3953). Bacterial inocula were prepared from fresh agar plates in Luria–Bertani medium (LB) and cultivated at 37 °C in an orbital shaker for 14 h. Then, the optical densities of the cultures were determined at 600 nm and serially diluted to the desired concentration. The number 4 × 10^−4^ colony forming units (CFU) of *S. Aureus* were inoculated into 1 mL of sterile 0.9% NaCl, in which the tested samples were immersed. After that, the samples were incubated at 24 °C for 3 h while gently shaking. Then, the samples were mixed and 20 μL drops of each sample were pipetted in five replicates onto LB agar plates, which were incubated at 37 °C for additional 16 h, after which, the number of CFU was counted and compared to the number of CFU on control plates (bacteria incubated only in 0.9% NaCl). The experiment was conducted under sterile conditions.

## 3. Results

**Figure 1:**
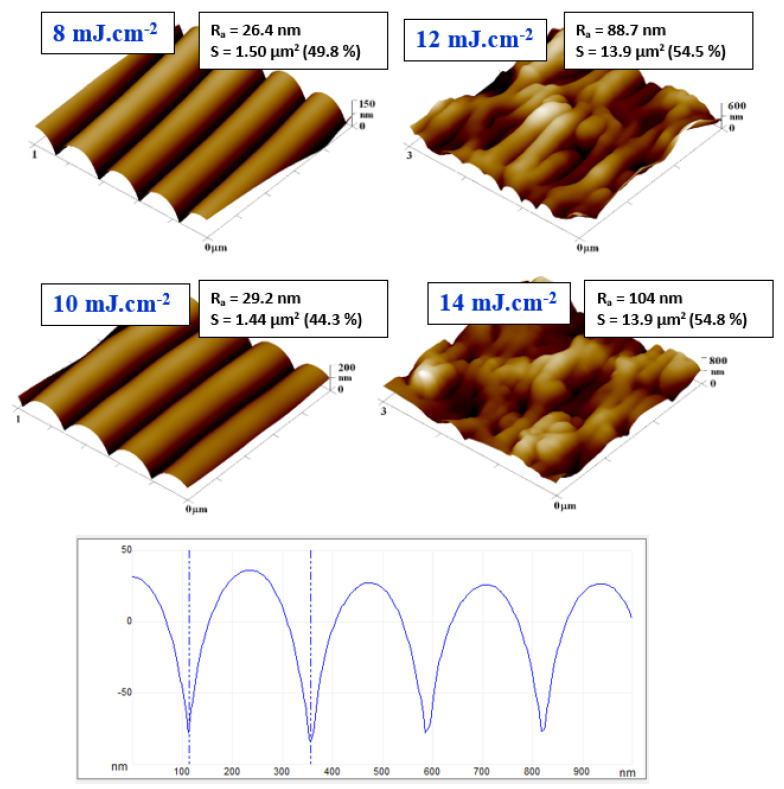
Surface morphology of laser-treated polystyrene foils doped with 5 wt. % of acetylsalicylic acid. The laser fluences from 8 to 14 mJ·cm^−2^ are introduced. The R_a_ value represents the arithmetic mean roughness, S represents an effective surface area with the difference form basic area in %. Bottom image represents line analysis of sample exposed with fluence of 10 mJ·cm^−2^.

**Figure 2:**
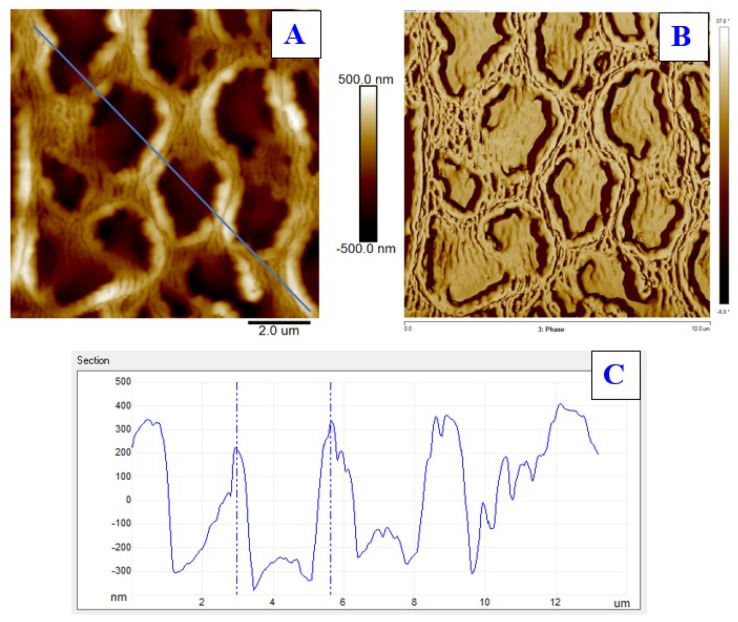
Surface morphology of laser-treated polystyrene foils doped with 5 wt. % of acetylsalicylic acid and laser fluence 16 mJ·cm^−2^ (**A**) and corresponding phase image (**B**). The R_a_ value is 167.0 nm, and S (effective surface area) is 137.0 μm^2^. Bottom image represents cross-section (**C**).

Surface morphology was examined both for doped PS foils and laser-treated PS foils. Due to incorporation of ASA into PS (both concentrations 5 and 10 wt. %), no significant surface changes were observed. Only a minor increase in surface roughness (up to 1 nm) was detected, therefore, these basic images were not introduced in the article. Even though it was previously confirmed that a PS foil is able to generate a periodic surface pattern due to excimer wavelength exposure, we had to verify this assumption for both ASA concentrations in PS prepared by the solvent casting method. As it is obvious from Figure 1, there exists a relatively narrow interval of excimer laser fluence, which can be applied for formation of a periodic pattern. For PS foils doped with 5% of ASA, the optimal laser fluence was 8 and 10 mJ·cm^−2^. The pattern width was approx. of 240 nm, while the pattern height of approx. 110 nm. The image of line analysis (one line from morphology data is extracted as x–z profile) is introduced also in Figure 1. The pattern formation was connected with a dramatic increase in both surface roughness and effective surface area. For example, for a doped PS foil with 5% of ASA, the dramatic increase in effective surface area from 1.05 to 1.45 μm^2^ was observed. A further increase in laser fluence led to partial collapse of the regular periodic pattern. This collapse was connected with a further increase in roughness and effective surface area which may also play an important role in antibacterial properties, which will be discussed further in this article. The increase in laser fluence up to 16 mJ·cm^−2^ induced a specific type of a PS pattern, which can be described as a honeycomb-like pattern (Figure 2), and was previously detected, e.g., for a biopolymer network prepared by an induced phase separation technique [32]. However, significant differences can be found, such as smaller dimension of a pattern unit and lower uniformity of the pattern structure. Another difference was found on the surface of hexagonal pattern units, at which some periodic structures still can be found, as it is documented on a phase image of the PS foil with 5% of ASA and treated with 16 mJ·cm^−2^.

Our primary focus was divided into two main aims. Firstly, if the laser exposure can be applied also for inducing of LIPSS on PS foils doped with ASA with a different amount of an active substance. These premise was based on our long-term experience of excimer exposure of polymeric foils, where we have exposed polymers such as polyethylene terephthalate (PET) [33], polyethylene naphthalate (PEN) [34], polyethersulphone (PES) [12] or study of Rebollar et al. aimed on polystyrene (PS) [35]. We have confirmed so far that for all of the aforementioned polymeric foils, it is possible to prepare linear periodic structures, if several conditions such as laser fluence, number of laser pulses and polarization of the beam is fulfilled. The second idea, which we will study in this article, is to determine if the pattern will exhibit any antibacterial properties.

### 3.1. Surface Morphology, Roughness and Surface Area

**Figure 3:**
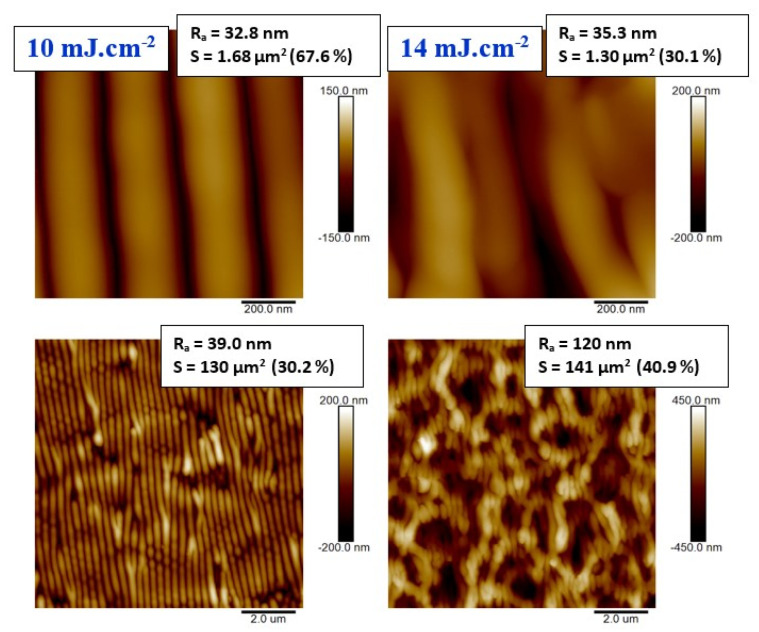
Surface morphology of laser-treated polystyrene foils doped with 10 wt. % of acetylsalicylic acid. The laser fluences of 10 and 14 mJ·cm^−2^ are introduced (two measured squares). The R_a_ value represents the arithmetic mean roughness, S represents an effective surface area with the difference from the plain area) in %.

**Figure 4:**
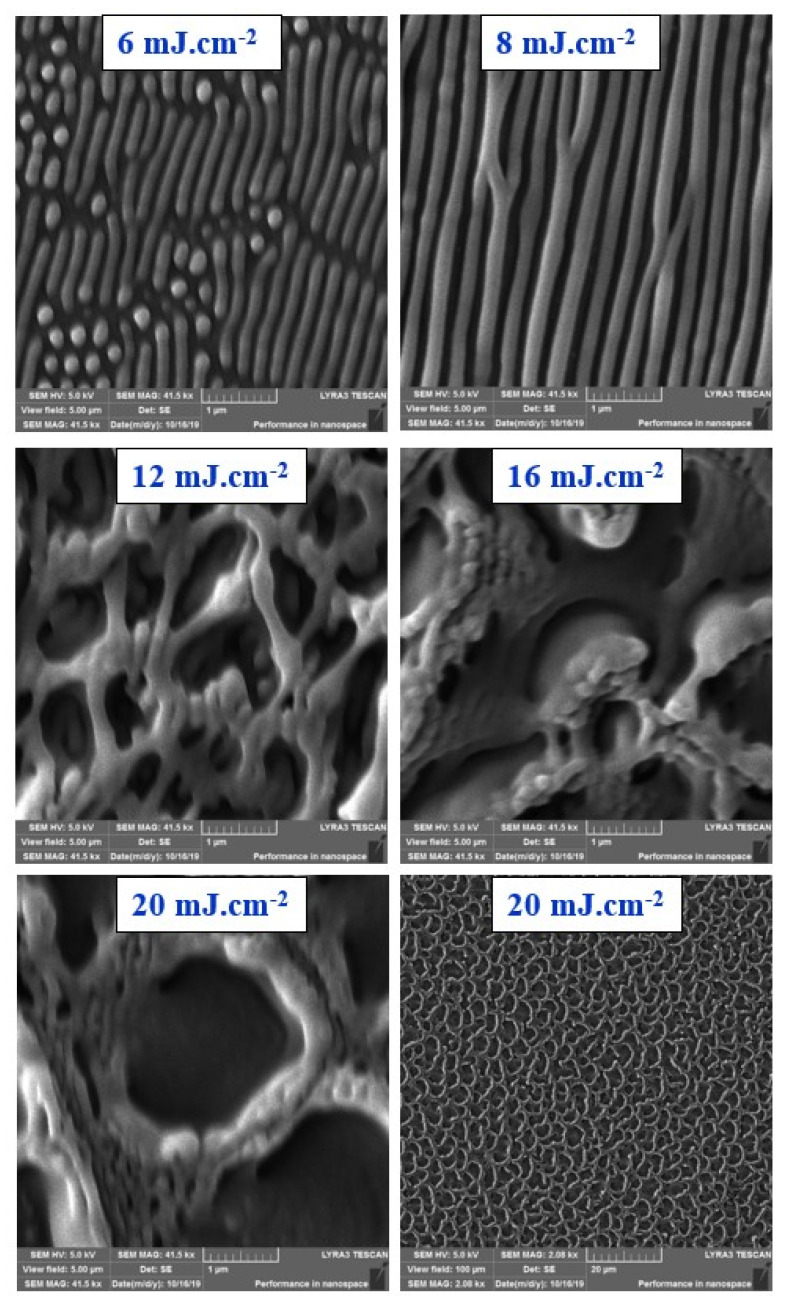
Images from scanning electron microscopy of laser-treated polystyrene foils doped with 5 wt. % of acetylsalicylic acid and laser fluence of 6–20 mJ·cm^−2^. The scanning area represents 5 × 5 μm^2^ and 100 × 100 μm^2^ (bottom right).

Similar behavior was confirmed for laser-treated PS foils doped with 10 wt. % of ASA, as documented in Figure 3. We have chosen to introduce detailed one micron square images of samples exposed to 10 and 14 mJ·cm^−2^ (upper images). Besides of that we have confirmed that the periodic regular pattern was also formed for exposure with laser fluence of 12 mJ·cm^−2^. For a better understanding of the surface pattern formation, also 10 × 10 μm^2^ were introduced for comparison. The collapse of a regular pattern was accomplished by a 200% increase in surface roughness when we compare 10 and 14 mJ·cm^−2^. It is evident that the complex process of the pattern formation, based on inhomogeneous surface energy distribution induced by surface wave and incoming wave interference [3], is not significantly disrupted by the addition of ASA into the PS. To confirm the acquired results, we also applied a surface morphology study with scanning electron microscope. The surface morphology images are shown in Figure 4 for laser exposure of PS foils with 5 wt. % of ASA. The particular stages of LIPSS formation are apparent at the initial stage of the pattern formation. For higher applied laser fluences, we have chosen a larger square on a sample (Figure 4, bottom line), on which the formation of structures similar to the honeycomb-like ones is well visible.

### 3.2. Surface Wettability

**Figure 5:**
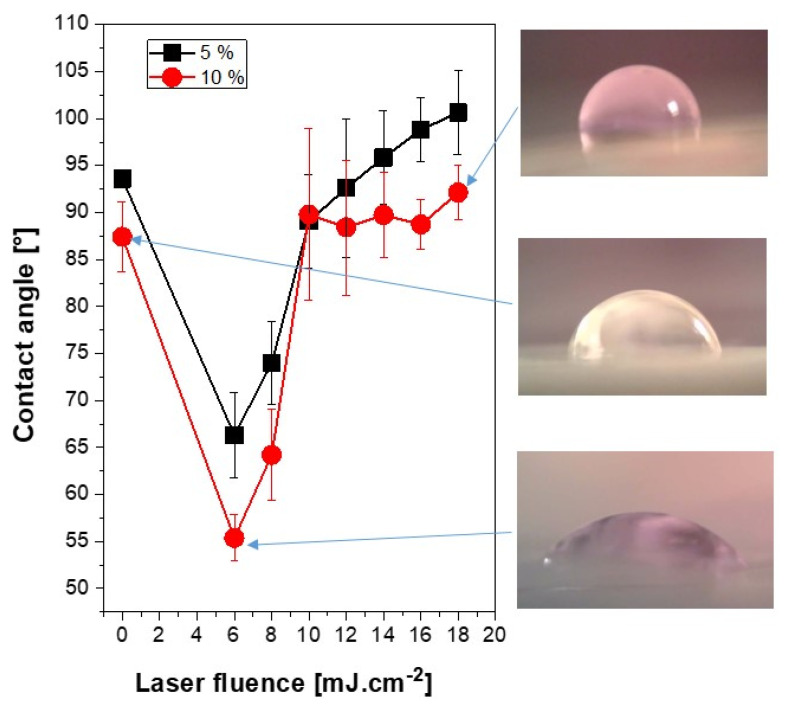
Water contact angle of polystyrene foils doped with 5 wt. % and 10% of acetylsalicylic acid and the same foils treated with excimer laser with laser fluence in the interval of 6–18 mJ·cm^−2^. Corresponding photos of selected water drops are introduced.

The well-known fact based on several published articles [36,37,38] is that changes in surface wettability and inducing of oxygen-containing groups may significantly affect both the antibacterial properties and biocompatibility of surfaces for selected cell lines. Cytocompatibility may be also affected by increased surface roughness [39]. The values of roughness may exceed hundreds of nanometers, so that the influence of surface morphology on wettability can also be the important factor [40]. Therefore, we have also aimed at the wettability of doped PS foils and the influence of laser treatment of doped PS foils on its wettability. The dependence of a contact angle on laser fluence is introduced in Figure 5. It is evident that doped PS with ASA exhibited relatively high values of contact angles, for 5% of ASA the surface was slightly hydrophobic, both foils exhibited values close to hydrophilicity/hydrophobicity edge value, 90°. As evident from Figure 5, excimer laser exposure induces significant increase of surface wettability for both PS foils (with 5% and 10% of ASA), the most pronounced change was observed for the lowest laser fluence, where a LIPSS formation can be detected. A decrease in a contact angle was more pronounced for PS doped with a higher amount of ASA (10%). However, from Figure 5, it is evident that for higher laser fluence the contact angle was stabilized, if the laser fluence is applied higher than 10 mJ·cm^−2^, and further for higher laser fluences the contact angles remain close to 90°. A slightly different situation was observed for PS foils doped with 5% of ASA, however, similarly as for foils doped with 10% of ASA a dramatic decrease in contact angles was observed for lower laser fluences.

The increase of laser fluence above 10 mJ·cm^−2^ induced a slight increase in a water contact angle, up to 100°, and the surface became slightly hydrophobic. The aforementioned behavior can be described by two different phenomena, firstly surface roughness and specific morphology [40]. The second phenomenon is a change in surface chemistry. This will be discussed in subsequent paragraphs and should play an important role both on wettability change, but also on antibacterial properties.

### 3.3. Surface Chemistry

**Figure 6:**
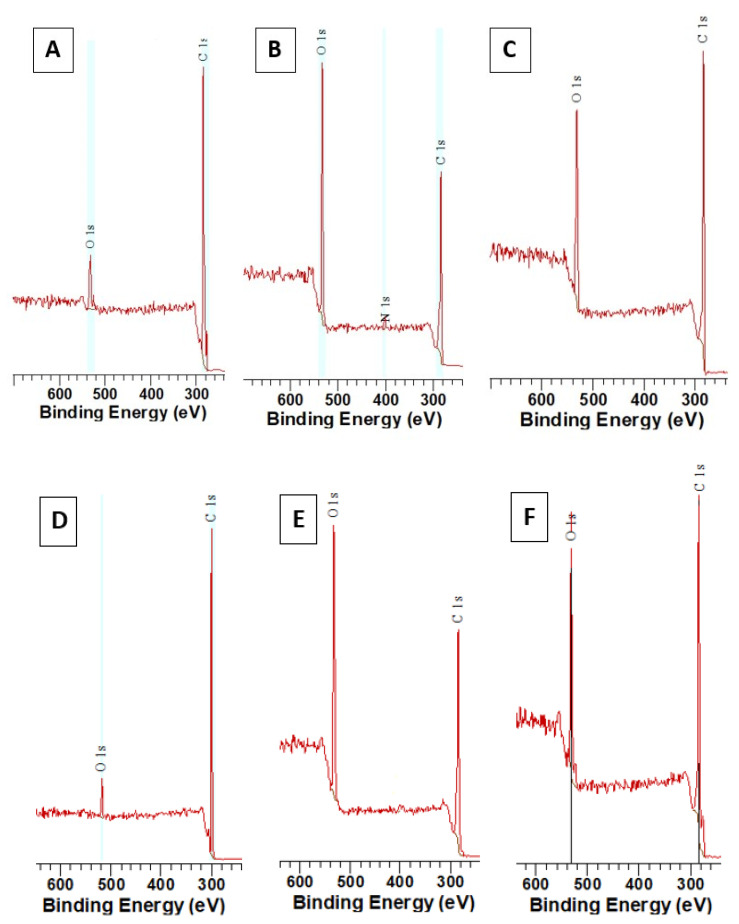
X-ray photoelectron spectroscopy (XPS) elemental spectra of three different situations regarding surface oxygen concentration on polystyrene. Polystyrene foil doped with 5 wt. % of acetylsalicylic acid (**A**), the same foil treated with laser fluence 8 mJ·cm^−2^ (**B**) and 14 mJ·cm^−2^ (**C**) are introduced. The same set of treatment is introduced for polystyrene doped with 10 wt. % of acetylsalicylic acid (**D**) and treated with 8 mJ·cm^−2^ (**E**) and 14 mJ·cm^−2^ (**F**).

**Figure 7:**
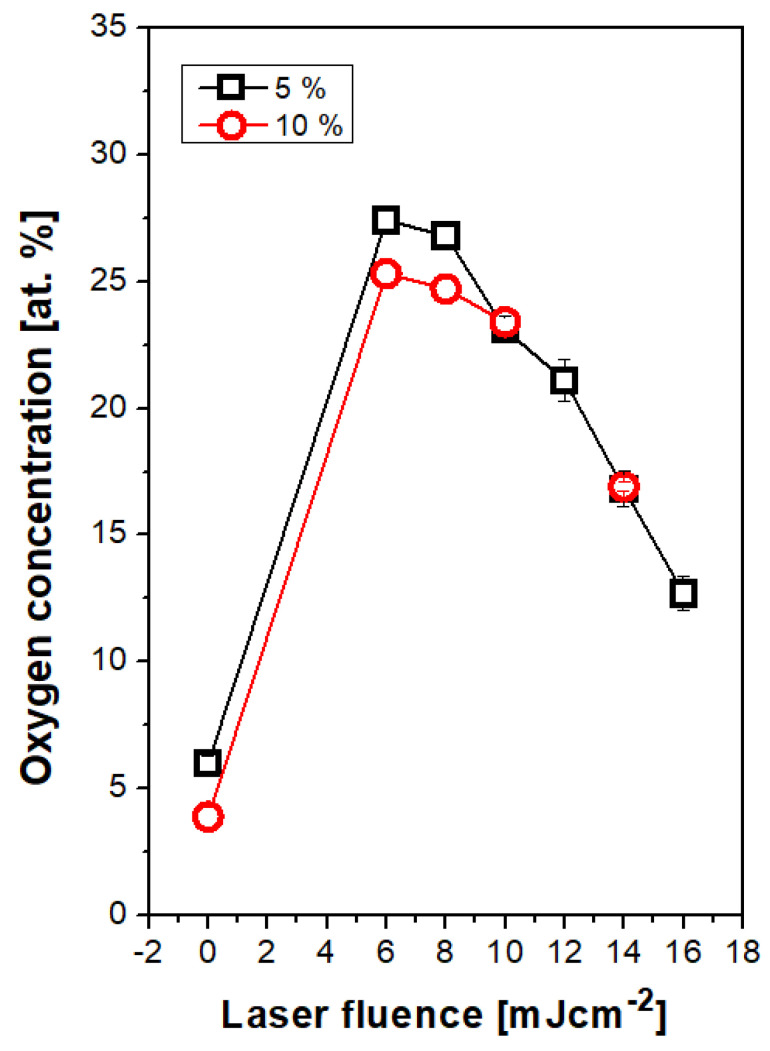
Dependence of atomic oxygen concentration on laser fluence determined with XPS method for polystyrene doped with acetylsalicylic acid (5 and 10 wt. %) and laser fluence in the interval of 6–16 mJ·cm^−2^.

Selected elemental spectra are introduced in Figure 6 for PS doped with 5 wt. % of ASA. We have selected three different “types” of surface chemistry, which will be discussed and were similarly observed also for PS foils with higher amount of ASA with an exception for treatment with 8 mJ·cm^−2^. Figure 6A represent spectrum of a pristine PS foil doped with 5% of ASA. As it is well documented in Figure 7, the amount of oxygen is approx. 5 at. %, due to incorporated ASA in the polymer structure. Surprisingly, a significant difference in surface oxygen concentration for PS foils doped with two different concentrations of ASA was not detected. Different situation was observed after laser exposure of doped PS foils with laser fluence 8 mJ·cm^−2^, where the optimal pattern formation was observed. Excimer treatment induced also an increase in oxygen concentration on polystyrene surface (Figure 7), for PS doped with 5% of ASA the increase was up to 27 at. % (Figure 7). The corresponding XPS spectrum is introduced in Figure 6B. The increase in oxygen concentration is connected with disruption of polymer bonds due to excimer exposure and free radical “hotspots”, where subsequent formation of oxygen-containing groups from the ambient atmosphere took place [41]. However, the process of pattern formation is connected with reorientation of macromolecular chains due to inhomogeneous distribution of excimer energy, as it was aforementioned. This process partially neglects the effect of an increase in surface oxygen, since the oxygen-containing groups are reoriented into the polymer bulk volume. This theory is confirmed well by the dependence of oxygen concentration on laser fluences above 10 mJ·cm^−2^ (Figure 7). A slight decrease in oxygen concentration with increasing laser fluence was observed, due to the process of pattern formation and for even higher laser fluences pattern collapse, a similar trend was maintained for this dependence both for ASA concentration 5% and 10% (in particular, corresponding XPS spectra are introduced in Figure 6D–F).

### 3.4. Antibacterial Properties

**Figure 8:**
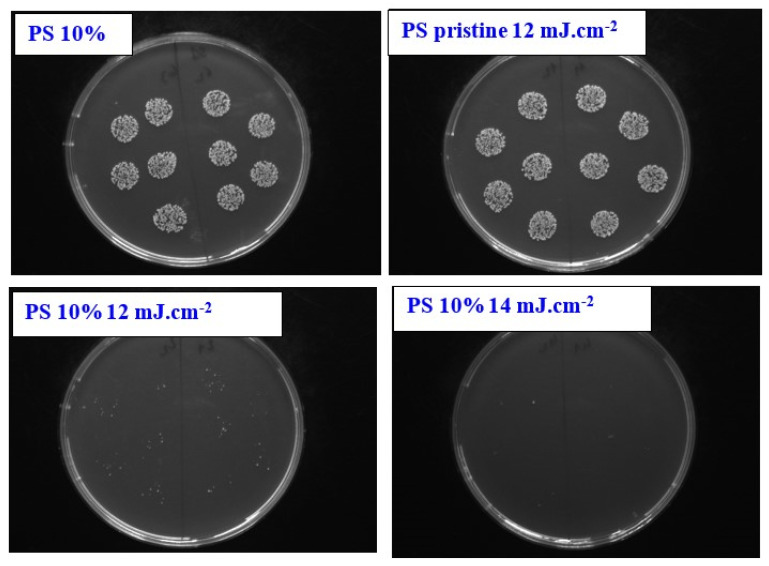
The photographs of *S. aureus* (CCM 3953) bacterial colonies after 3 h of bacterial growth, the following samples are introduced: polystyrene doped with 10 wt. % of acetylsalicylic acid (PS 10%), pristine polystyrene exposed with 12 mJ·cm^−2^; polystyrene doped with 10 wt. % of acetylsalicylic acid and exposed with 12 mJ·cm^−2^ (PS 10% 12 mJ·cm^−2^) and polystyrene doped with 10 wt. % of acetylsalicylic acid and exposed with 14 mJ·cm^−2^ (PS 10% 14 mJ·cm^−2^).

Selected PS foils were tested on antibacterial response. Gram-positive bacteria strain *of S. aureus* was selected for evaluation. The results of antibacterial testing represent the photographs of CFU, as it is evident from Figure 8. We supposed that the ASA itself may serve as an antibacterial agent, before the experiments. Surprisingly, as it can be seen on Figure 8, only the doping itself does not lead to significant changes in antibacterial properties of PS. Similar results were obtained only for laser-treated PS, which was subsequently treated with an excimer laser. A significantly different situation was observed for a combination of laser exposure and addition of ASA into a PS film. From bottom images of Figure 8, it is evident that the laser treatment of PS doped with ASA film exhibited outstanding antibacterial results. By such approach the excellent antibacterial surface was prepared, where the number of bacterial colonies (CFU, colony forming units) decreased, to almost zero in case of 12 mJ·cm^−2^ (the same result was observed for laser treatment with 10 mJ·cm^−2^, where the periodic pattern is formed) and zero in case of laser treatment with 14 mJ·cm^−2^ (the same result was observed for treatment with 16 mJ·cm^−2^).

## 4. Conclusions

The stable PS foils with two different concentration of ASA (5 and 10 wt. %) were successfully prepared by the solvent-casting method. The doped PS foils were subsequently treated with an excimer laser. We confirmed that doped PS foils with ASA can be used for construction of laser-induced periodic surface structures (LIPSS). By exposure of polymers with lower and higher concentration, the pattern exhibited regular shape in a narrow interval of the laser fluence, from 8 to 10 mJ·cm^−2^. By the collapse of a periodic pattern, we have prepared so called honeycomb-like structure on PS by selected laser exposure (16 mJ·cm^−2^ and higher). The previous aim was to prepare antibacterial surface by simple addition of ASA. This was not successful, so we proceeded with surface modification based excimer laser exposure. The periodic pattern induced by excimer laser in combination with the unique surface morphology was confirmed to enhance the antibacterial properties significantly compared to surface without the presence of LIPSS. This effect was achieved mainly due to the increased effective surface area of the pattern and periodic/honeycomb surface morphology. Also, the excimer beam effectively enhanced the surface chemistry and supported the release of ASA from partially disrupted polymer surface. The laser exposure induced significant changes in surface wettability and chemistry of doped PS. The wettability was significantly increased for lower laser fluences. Exposure with higher laser fluences, which even induced significant changes in surface chemistry, did not significantly alter the material surface wettability (contact angle was of ca. 90°). The addition of ASA increased the oxygen concentration in PS films, which was dramatically increased by laser exposure, mostly for low laser fluence doses. Laser treatment of ASA-doped PS films exhibited outstanding antibacterial properties. The technique of a combination of the excimer laser and doping of PS films led to a decrease in the number of CFU to zero for Gram-positive bacteria of *S. aureus*.
